# Supplementary material for: Gut Microbiota Serves a Predictable Outcome of Short-Term Low-Carbohydrate Diet (LCD) Intervention for Patients with Obesity
Source: Microbiol Spectr. 2021 Sep 15;9(2):e00223-21. doi: 10.1128/Spectrum.00223-21 (PMC8557869; doi:10.1128/Spectrum.00223-21)
Supplement: SUPPLEMENTAL FILE 2 — Supplemental material. Download Spectrum.00223-21-s0002.pdf, PDF file, 2.3 MB [file spectrum.00223-21-s0002.pdf]

Gut microbiota serves a predictable outcome of short-term low-carbohydrate diet (LCD) intervention for patients with obesity

Susu Zhang[1,2,#], Peili Wu[1,4,#], Ye Tian[1,2, #], Bingdong Liu[2,3,#],Liujing Huang[2], Zhihong Liu[2], Nie Lin[1,5], Ningning Xu[1], Yuting Ruan[1], Zhen Zhang[1], Ming Wang[6], Zongbing Cui[2], HongWei Zhou[7], Liwei Xie[1,2,8,*], Hong Chen[1,*], Jia Sun[1,*]

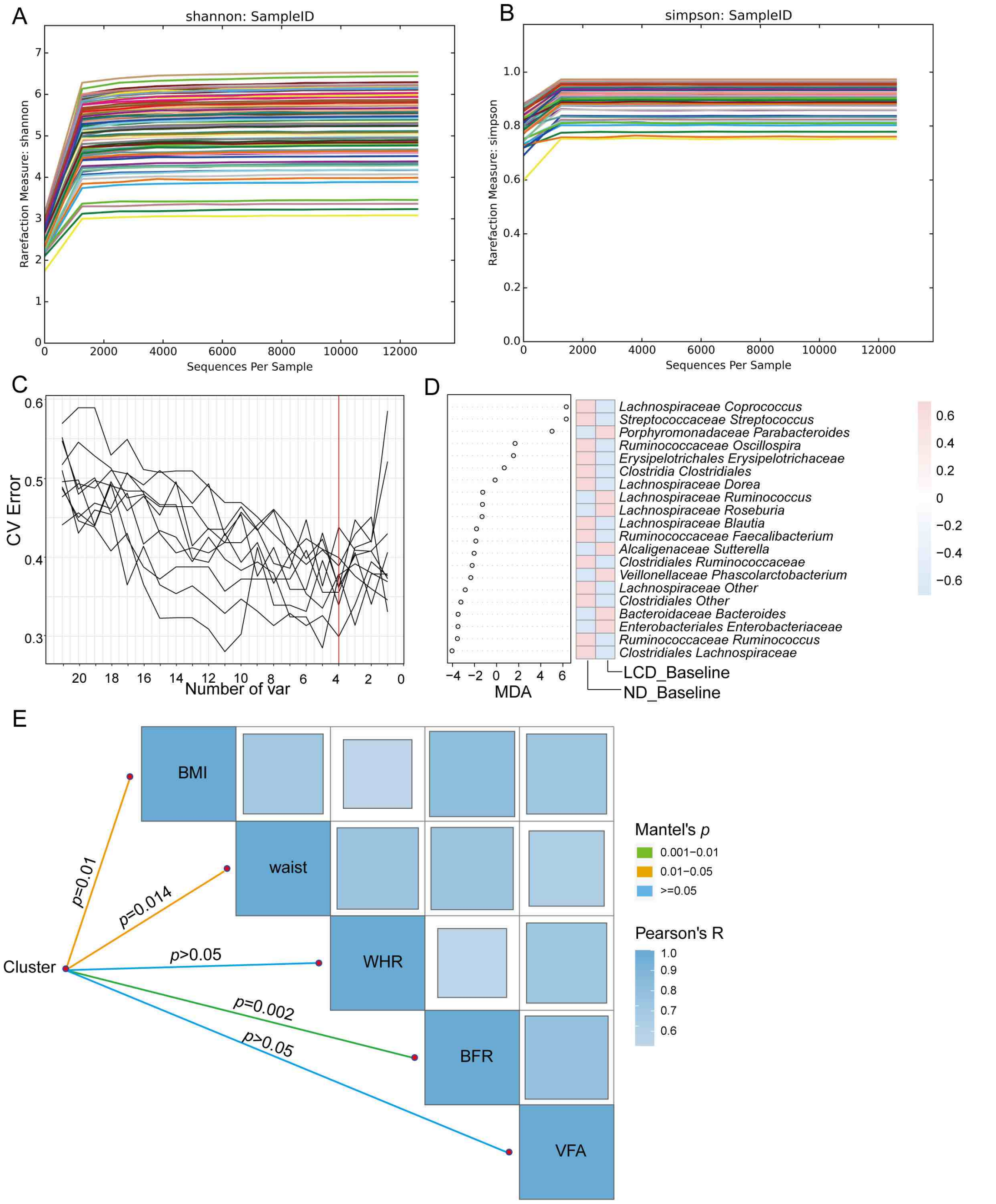

Supplementary Figure S1 is related to Figure 2. Information about rarefaction measurement and random forest of ND and LCD group at baseline. A-B) Rarefaction measurement of Shannon (A) and Simpson(B) index presented a saturate platform and indicated sequencing depth was enough to capture all bacterial species and qualified for

downstream analysis. (C) Four bacterial markers at the genus level were selected as optimal biomarkers of the random forest model between the ND group and LCD group at baseline. (D) The relative abundance of each bacteria at the genus level in the predictive model was assessed by MDA. The heatmap illustrated the comparison of bacteria filtered by random forest via 5-fold cross-validation in the ND group and LCD group at baseline. (E) Matrix correlation between change of relative abundance after LCD intervention and clinical parameter (BMI, waist, WHR, BFR and VFA).

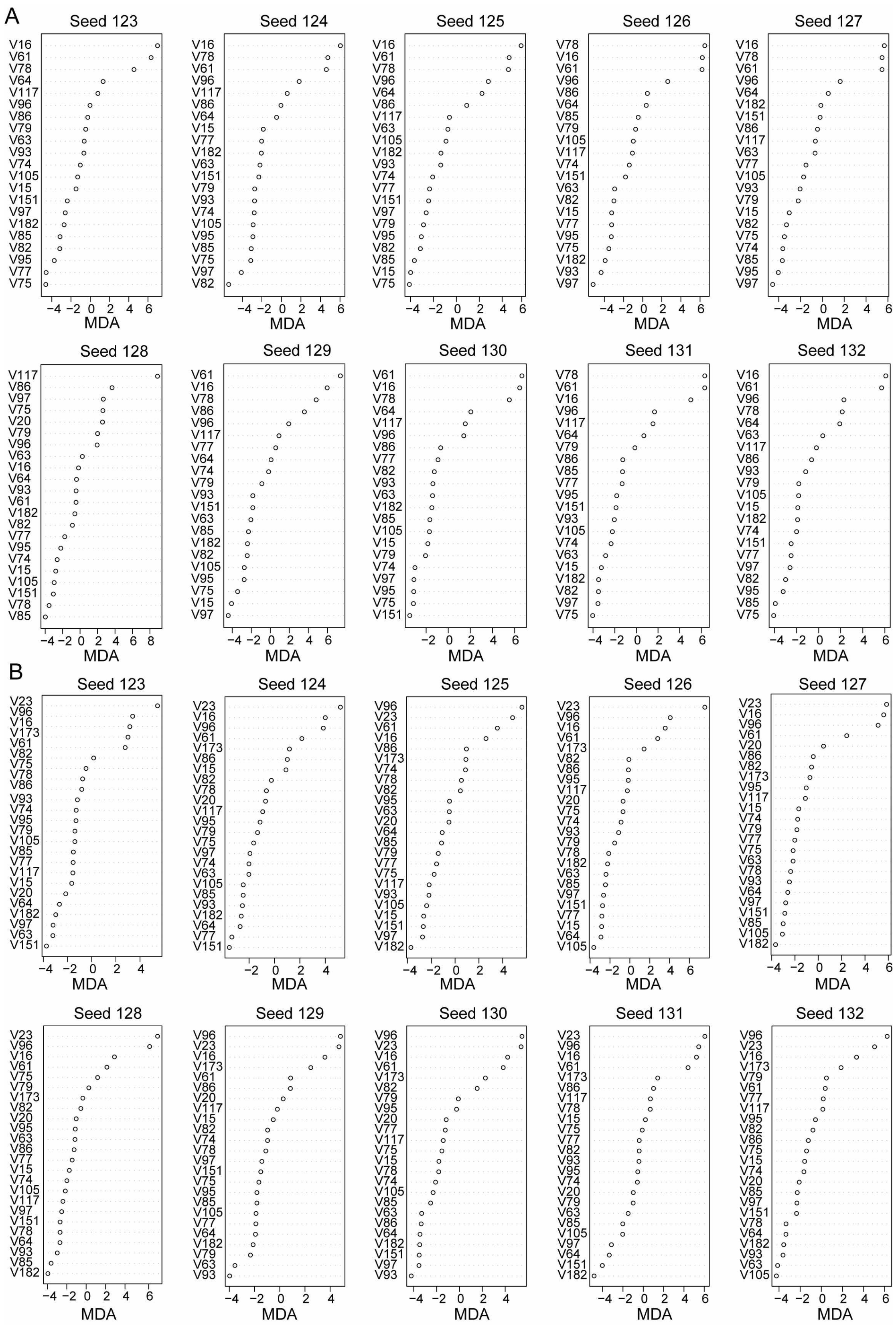

Supplementary Figure S2 is related to Figure 2. Detailed results of random forest in ten different random seed for ND group and LCD group at two different time points. (A-B) Detailed results of 10 trials of random forest algorithm for ND group and LCD

group at baseline stage (A) and end-stage (B). The bacteria in each predictive model were assessed through the MDA at the genus level.

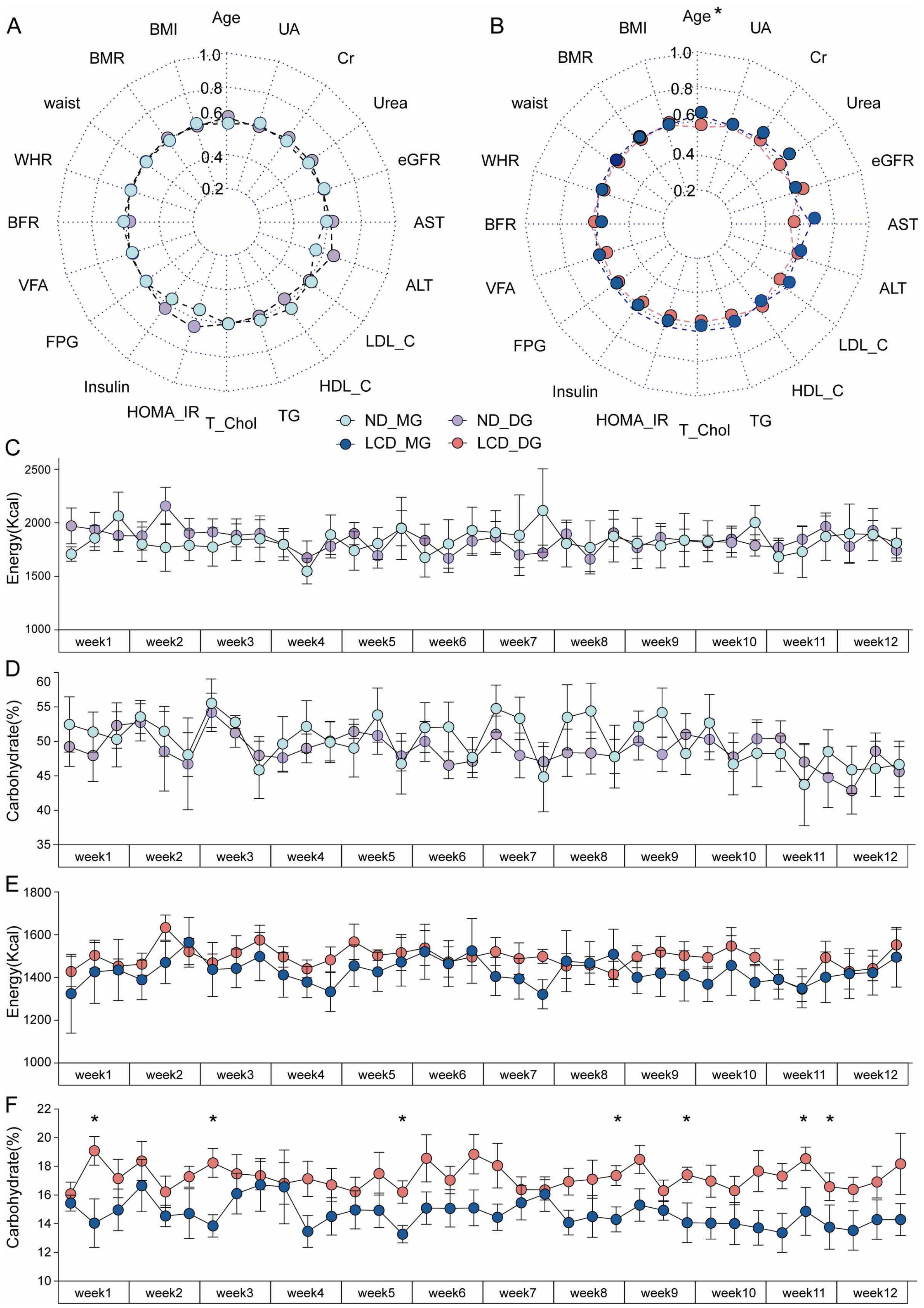

Supplementary Figure S3 is related to Figure 3. Clinical information of subgroups in two different dietary groups. (A-B) Radar plots of baseline clinical characterizations in ND subgroups (A) and LCD subgroups (B). Data are expressed as mean, *$p < 0.05$ is from unpaired, two-sided Student's *t*-test. (C, E) Mean energy calculated from food

conversion according to 24 h dietary recalls of 3 days in every week was almost the same in ND subgroups (C) and in LCD subgroups (E) (p>0.05 for each time-point, are from unpaired, two-sided Student's t-test. (D, F) Mean proportions of carbohydrates in ND subgroups (D) and LCD subgroups (F) calculated from 24 h dietary recalls of 3 days in every week. Data are expressed as mean ± SEM, *$p < 0.05$ are from unpaired, two-sided Student's t-test.

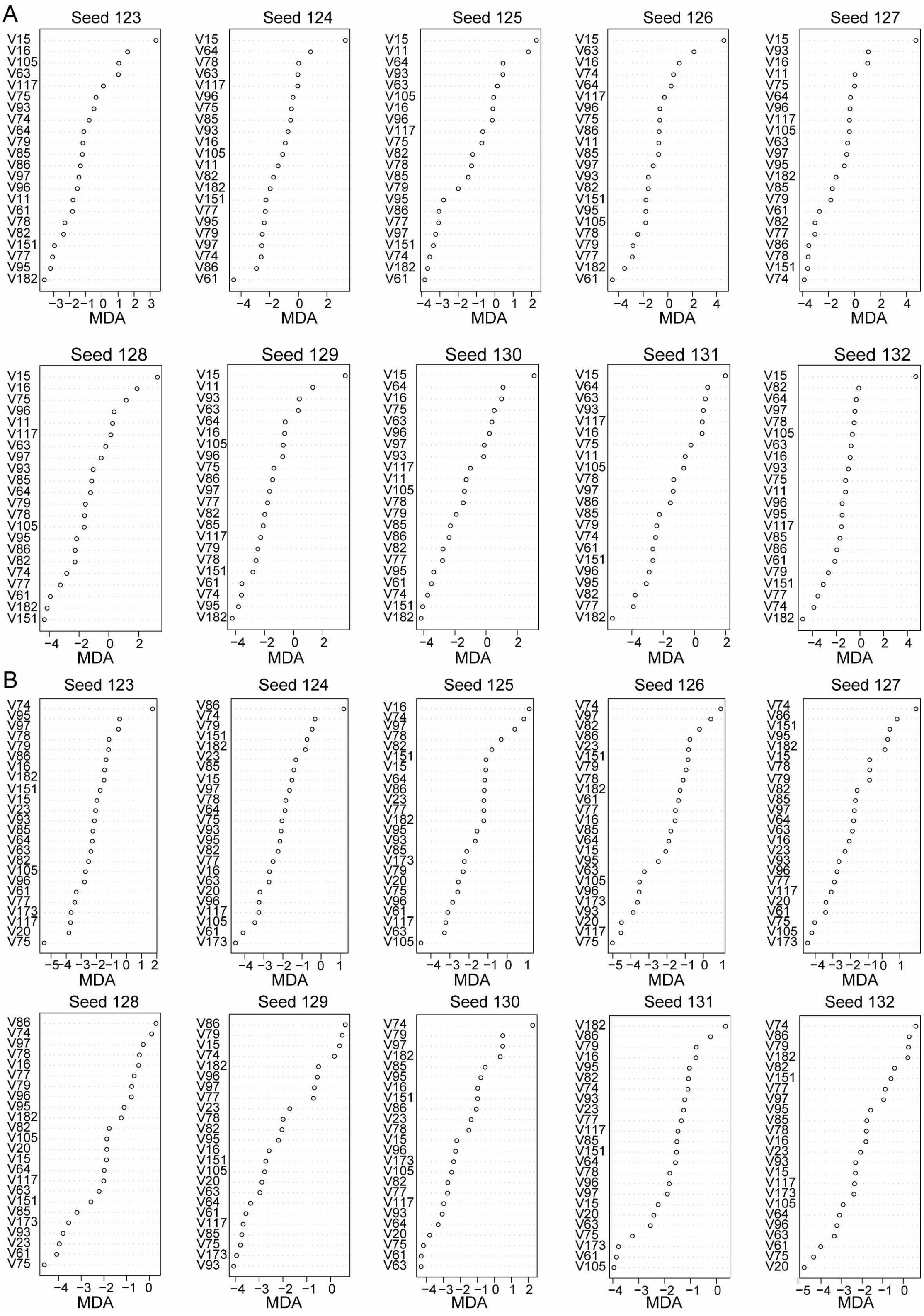

Supplementary Figure S4 is related to Figure 4. Detailed results of random forest in ten different random seed for LCD subgroup at two different time points. (A-B) Detailed results of 10 trials of random forest algorithm for LCD subgroups at baseline stage (A) and end-stage (B). The bacteria in each predictive model were assessed

through the MDA at the genus level.

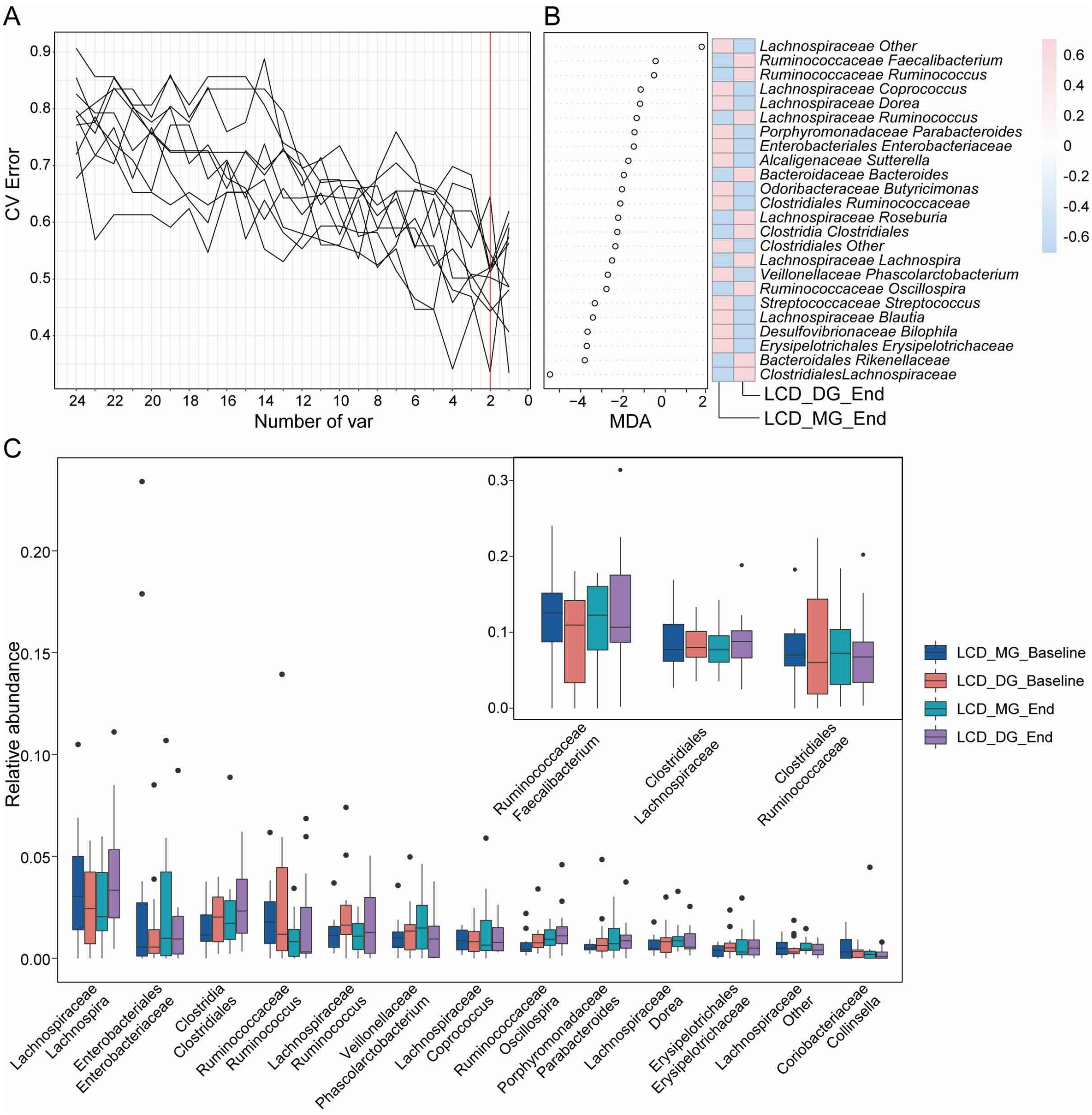

Supplementary Figure S5 is related to Figure 4. Optimal random forest model of LCD subgroups at end-stage and all union optimal bacterial biomarkers selected by the random forest. (A) Two bacterial markers at the genus level were selected as optimal biomarkers of the random forest model between LCD subgroups after 12 weeks of LCD intervention. (B) The relative abundance of each bacteria at the genus level in the predictive model was assessed by MDA. The heatmap illustrated the comparison of bacteria filtered by random forest via 5-fold cross-validation in LCD subgroups at the end-stage. (C) Box plots showed no statistically significant difference of all union optimal bacterial biomarkers selected through the random forest algorithm at baseline stage and end-stage. Two-way ANOVA with repeated measures followed by a Tukey post hoc test was used to compare the relative abundance of LCD_MG and LCD_DG

at these two time points using GraphPad Prism 8.0.2.
